# The swimming habits of women who cold water swim

**DOI:** 10.1177/17455057241265080

**Published:** 2024-08-21

**Authors:** Megan Pound, Heather Massey, Sasha Roseneil, Ruth Williamson, Mark Harper, Mike Tipton, Jill Shawe, Malika Felton, Joyce Harper

**Affiliations:** 1 Institute for Women’s Health, University College London, London, UK; 2 Department of Sport, Health and Exercise Science, University of Portsmouth, Brighton, UK; 3 University of Sussex, Brighton, UK; 4 Hampshire Hospitals NHS Foundation Trust, Hampshire, UK; 5 University Hospitals Sussex NHS Foundation Trust, Worthing, UK; 6 Sørlandet Sykehus, Kristiansand, Norway; 7 University of Plymouth and Royal Cornwall Hospitals NHS Trust, Cornwall, UK; 8 Department of Rehabilitation & Sport Sciences, Bournemouth University, Poole, UK

**Keywords:** cold water swimming, habits, swimming, wild swimming, women, women’s health

## Abstract

**Background:**

Cold water swimming is growing in popularity, especially among women. We have previously reported that women felt that cold water swimming helps with their menstrual and menopause symptoms. But little is known about the habits of women who cold water swim.

**Objectives:**

To determine the habits of women who cold water swim.

**Design:**

This was a mixed-methods study.

**Methods:**

An online survey asked women who cold water swim about their experience of swimming and how this affected their menstrual and menopause symptoms. The survey was advertised for 2 months on social media, with a focus on advertising in cold water swimming Facebook groups. In this article, only the questions on the women’s swimming habits were analyzed.

**Results:**

The analysis of 1114 women, mainly from the United Kingdom, revealed that most had been swimming for 1–5 years (79.5%). Most swim in the sea (64.4%), and only 15.5% swim alone. The majority (89.0%) swim all year around, swimming for mainly 30–60 min in the summer and 5–15 min in the winter. The women mostly swim wearing swimming costumes (skins) throughout the year. The majority of the free-text responses showed women found mental and physical benefits from cold water swimming.

**Conclusion:**

It was not surprising to learn that women swim for longer in the summer than the winter, but hearing how they feel cold water swimming helps their physical and mental health is important. With the limitations on access and safety of many wild swimming sites in the United Kingdom, it is time to ensure that cold water swimming is safer and more supported.

## Introduction

Cold water swimming is increasing in popularity in the United Kingdom and abroad, with membership of the UK Outdoor Swimming Society increasing from 300 members in 2006 to 187,000 members in 2022.^1,2^ In the current literature, there is some evidence that voluntary immersion in cold water may have health benefits.^1,3,4^ However, most data remain anecdotal or come from case studies.^5,6^

Cold water swimming has a multitude of definitions. The International Ice Swimming Associations (IISA) and the International Winter Swimming Association (IWSA) define cold water as +5.1 to +9°C, freezing water as +2.1°C to +5°C and ice water as −2°C to 2°C.^7^ However, many studies use cold water swimming as an umbrella term to describe swimming at temperatures up to 20°C throughout the year.^8^ Not all cold water swimming consists of significant swimming, but rather cold water immersion (CWI), or colloquially ‘cold plunges’ or ‘dipping’, where swimmers expose themselves to the effect of the cold water for a short period of time.^9,10^

## Health benefits of cold water swimming

The physical and mental benefits of swimming have been long established.^1,3,4,5,6,8^ Swim England have collaborated with the Royal College of General Practitioners (RCGP) to promote ‘swimming as medicine’.^11^

The aerobic benefits of swimming are potentially enhanced by the effect of cold water.^12^ Ice baths or CWI are frequently used to aid athletes’ recovery and muscle repair^13^ but their actual benefits are debated.^14–16^ Increasingly, studies are demonstrating the adaptations that cold water swimmers develop to increase their cold water tolerance, as well as further endocrine, cardiovascular and psychological benefits.^1,10^

Massey et al.^17^ undertook a survey of open water swimmers and showed the positive perceived impact on mental health conditions as well as suggesting a potential long-term improvement in the mood of novice cold water swimmers. Case studies suggest that cold water swimming could be used as treatment for depression associated with chronic illness and major depressive disorder.^5,18,19^

Being present in an outside or ‘blue’ space has been shown to have a positive impact on mental health, following the theory of attention restoration, promoting recovery from cognitive exhaustion.^19,20^ Following a series of interviews conducted with participants while swimming, Denton and Aranda^21^ concluded that the impact is multi-layered and included transformation, reorientation and connection. On a physiological level, it has been hypothesized the parasympathetic nervous system promotes this restoration, stimulating a stress recovery response.^22^ More recently, Burlingham et al.^3^ undertook a feasibility trial of cold water swimming in which it was observed that symptoms of depression and anxiety were reduced in participants.

## Risks of cold water swimming

There are risks of cold water swimming including the obvious risk of drowning and the risk of cardiac arrest which is exacerbated by ‘cold shock’.^23,24^ Local neuromuscular cooling can lead to physical incapacitation in as little as 10 min.^25,26^ Hypothermia is also a risk, especially for experienced, cold-adapted swimmers, who are poor at accurately estimating their deep body temperature while swimming.^27^ Therefore, there is a danger that they may be unable to determine when to exit the water before becoming hypothermic.^27^ Swimming-induced pulmonary oedema (SIPE) occurs in healthy individuals as a result of cold water swimming.^28^ It is characterized by acute onset of cough and dyspnoea while swimming, sometimes accompanied by exhaustion, excessive sputum and haemoptysis, or a combination.^28^

There is also the issue of pollution. Sewage pollution is becoming an increasingly common concern in UK rivers and seas, increasing the likelihood of gastroenteritis and other infections.^29,30^ In the United Kingdom, designated bathing waters are typically only monitored during the bathing season, generally from May to September. Although most UK designated bathing waters meet prescribed standards during the bathing season, this may not always be the case.

With the increase in popularity of cold water swimming, we felt it was important to study women who cold water swim as no studies have been conducted specifically with women. The survey we did was split into two studies: the habits of cold water swimmers are reported here and questions relating to whether women feel that cold water swimming affects women’s menstrual and menopause symptoms, which has been reported elsewhere.^31^ Through this, we hope to characterize those that engage in cold water swimming and gain a greater understanding of their motivations and hurdles to swimming.

## Methods

This was a mixed-methods, observational study. This project was approved by University College London (UCL) Research Ethics Committee: 9831/007. To undertake the survey, participants had to click consent to continue onto the survey questions. The Strengthening the Reporting of Observational studies in Epidemiology (STROBE) Guidelines were followed when preparing this article.

A research team of academic and clinical experienced cold water swimmers (Joyce Harper, Sasha Roseneil, Ruth Williamson, Heather Massey) designed the online survey using Qualtrics, with discussion with additional cold water swimmers. The survey comprised 42 questions which were mainly multiple choice with some open questions (Supplemental Material 1).

The survey was validated through in-depth interviews with six women who were cold water swimmers, going through each question in turn. They made minor edits and suggestions. The final survey was advertised mainly via cold water swimmers Facebook groups but was also promoted on social media (Supplemental Material 2). The survey was live from 7 June to 6 August 2022 and was closed when it felt that saturation was reached, that is no additional insights were obtained.

Participants were considered eligible if they were women who swim outdoors in unheated water. The survey excluded men and those who did not swim outdoors in unheated water. After consenting to undertake the survey, the women were asked descriptive data including their age, sexual orientation, relationship status, number of children and self-described health data. They were asked about their cold water swimming habits and how swimming affected any menstrual and menopausal symptoms (the latter data are not shown in this article and have been published elsewhere.^31^

## Results

### Demographics

In total, 1357 women started the survey and 1114 pressed submit (1114/1357, 82.1%). Only submitted responses were included in the study.

The overwhelming majority of the participants resided in the United Kingdom (1041/1114, 93.4%; Table 1). The age of the participants ranged from 16 to 80 years, with an average age of 49 years. Most of the participants were aged between 45–54 years (577/1114, 51.7%), heterosexual (981/1114, 88.1%) and married/in a civil partnership (670/1114, 60.1%). Nearly 70% of participants had one or more children (774/1114, 69.5%), but a large proportion did not (340/1114, 30.5%).

**Table 1.** Personal information of the women that completed the survey.

Residence	*n*	%
UK	1041	93.4
Other	73	6.6
**Age**		
<20	2	0.2
20–24	7	0.6
25–29	23	2.1
30–34	45	4.0
35–39	50	4.5
40–44	144	12.9
45–49	271	24.3
50–54	306	27.5
55–59	182	16.3
60–64	64	5.7
65–69	17	1.5
70–74	2	0.2
>75	1	0.1
**Sexuality**		
Heterosexual	981	88.1
Bisexual	55	4.9
Lesbian/gay	30	2.7
Asexual	5	0.4
Pansexual	15	1.3
Prefer not to say	28	2.5
**Relationship status**		
In a relationship not cohabiting	65	5.8
In a relationship cohabiting	180	16.2
Single	167	15.0
Married/civil partnership	670	60.1
Widowed	10	0.9
Other—in your own words	15	1.3
Prefer not to say	7	0.6
**Number of children**		
1	158	14.2
2	426	38.2
3	139	12.5
4 or more	47	4.2
I do not have children	340	30.5
Prefer not to say	4	0.4

Participants tended to have either university undergraduate (303/1114, 27.2%) or university undergraduate and postgraduate (555/1114, 49.8%) qualifications (Table 2). Over half of the women had no religion (601/1114, 53.9%), and the vast majority had no disability (927/1114, 83.2%). The overwhelming majority of the participants described themselves of White (English/Welsh/Scottish/Northern Irish/British) background (965, 86.6%).

**Table 2.** Demographics of the women that completed the survey.

Education	*n*	%
Secondary school	51	4.6
A level/College level	153	13.7
University undergraduate	303	27.2
University postgraduate	555	49.8
Other	48	4.3
Prefer not to say	4	0.4
**Religion**		
Christian	388	34.8
Muslim	2	0.2
Jewish	9	0.8
Hindu	1	0.1
Sikh	1	0.1
Buddhist	22	2.0
No religion	601	53.9
Any other	62	5.6
Prefer not to say	28	2.5
**Disability**		
No disability	927	83.2
Sensory impaired	19	1.7
Physically/mobility impaired	22	2.0
Specific learning difficulty or disability	36	3.2
General learning disability (cognitive)	2	0.2
Long-term illness or health condition	102	9.2
Autism spectrum disorder	27	2.4
Other	45	4.0
Prefer not to say	2	0.2
**Ethnicity**		
White–English/Welsh/Scottish/Northern Irish/British	965	86.6
White Irish	38	3.4
Any other White background	97	8.7
Asian/Asian British Indian	4	0.4
Asian/Asian British Pakistani	1	0.1
Any other Asian background	3	0.3
Black/Black British African	0	0.0
Black/Black British Caribbean	0	0.0
Any other Black/African/Caribbean background	0	0.0
Latino	2	0.2
Mixed ethnic background	18	1.6
Any other ethnic group	1	0.1
Prefer not to say	4	0.4

### Health of the participants

**Figure 1.**
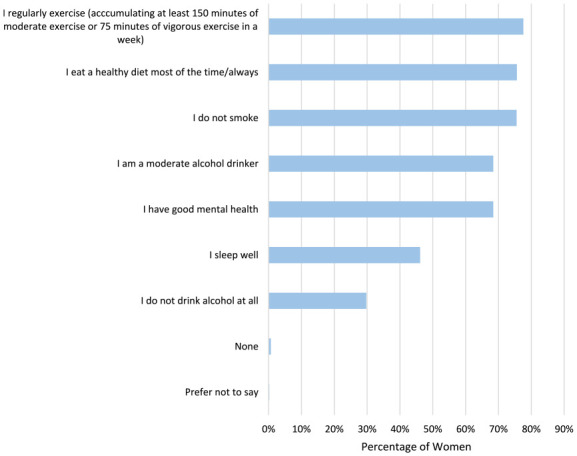
Women were asked to describe their health by selecting what statements applied to them.

Women were asked to describe their health by selecting statements they felt applied to them (Figure 1). Most women stated they regularly exercise (accumulating at least 150 min of moderate exercise or 75 min of vigorous exercise in a week) (866/1114, 77.6%), had a healthy diet (participants’ own perception of ‘I eat well most/all of the time’) (844/1114, 75.6%) and do not smoke (843/1114, 75.5%). Only 68.4% (577/1114) stated they would consider themselves to have good mental health.

**Figure 2.**
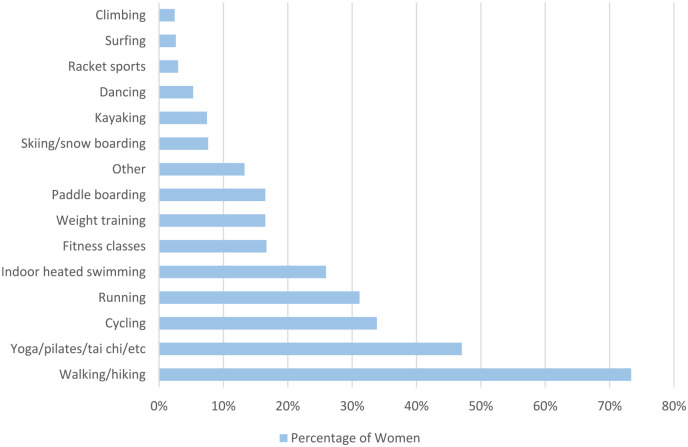
Women were asked to describe the exercise they participate in outside of cold water swimming.

The women were asked to select the type of exercise they participated in outside of cold water swimming (Figure 2). The most common exercises stated were walking/hiking (817/1114, 73.3%) and yoga/Pilates/tai chi (524, 47.0%).

### Cold water swimming habits

Women were asked about their cold water swimming habits.

**Figure 3.**
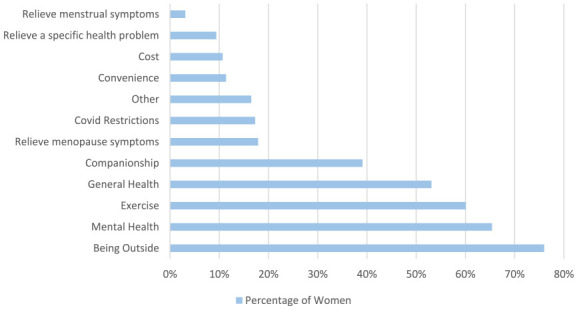
Women were asked the main reasons they choose to cold water swim.

Women were asked why they swim, selecting all answers that applied (Figure 3). The majority of women (847/1114, 76.0%) stated the main reason they swim was to be outside, followed by their mental health (729/1114, 65.4%). Of the 729 women who stated one of the main reasons they swim is for their mental health, 299 stated they have good mental health, and 430 did not state they have good mental health. They also stated that exercise (670/1114, 60.1%) and general health (592/1114, 53.1%) were their main reasons for swimming. Nearly one in five women (199/1114, 17.9%) swim mainly to relieve menopause symptoms, but only 3.1% (35/1114 women) swam mainly to relieve menstrual symptoms.

We did not specifically ask women if they were perimenopausal, postmenopausal and so on, but 785 women were considered menopausal and directed to finish the menopause section, compared with 711 menstrual. Some women would be considered as both as they would have been going through the menopause during their time in cold water swimming.

**Figure 4.**
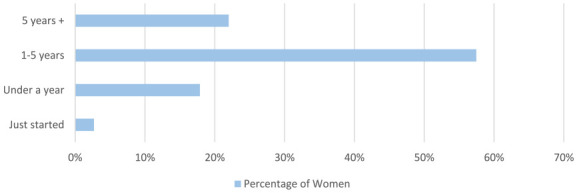
Women were asked how long they have been cold water swimming.

Women were asked how long they have been in cold water swimming (Figure 4). The majority of women had been swimming for more than a year (885/1114, 79.5%).

**Figure 5.**
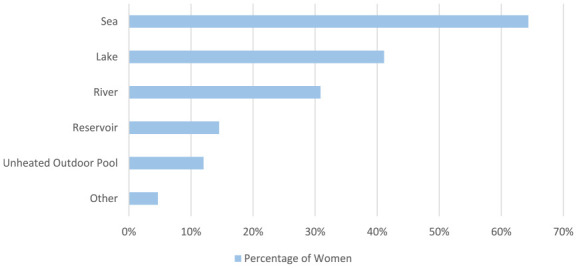
Women were asked in which types of water they usually cold water swam in, ticking all that applied.

**Figure 6.**
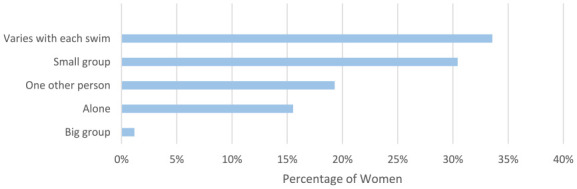
Women were asked who they normally swim with.

Women were asked in which types of water they swim in (Figure 5), and who they swim with (Figure 6). Most of the women swim in the sea (717/1114, 64.4%), followed by a lake (458/1114, 41.1%). Most women swim with other people, with just 15.5% (173/1114) stating they normally swim alone.

The women were asked to state whether they swim in the summer, winter or both (Table 3). In total, 10.6% (118/1114) of women stated they swim in the summer only, compared with 5/1114 (0.4%) stating they only swim in the winter. The majority (991/1114, 89.0%) stated they swim in both the summer and winter. For the following graphs, the summer swimming statistics are out of 1109, compared with the winter swimming statistics which are out of 996.

**Table 3.** Women were asked what season they choose to swim.

When swim?	Number	%
Summer only	118	10.6
Winter only	5	0.4
Summer and winter	991	89.0

**Figure 7.**
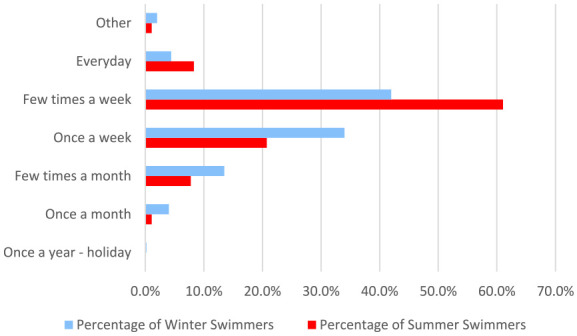
A comparison of how often women swim in the summer versus the winter.

Women were asked how often they choose to swim in each season separately (Figure 7). Those who stated they swim in both summer and winter completed the summer and winter question separately. In summer, the most common frequency for swimming was a few times per week (677/1109, 61.7%). Of the 996 women that stated they swim in the winter, most swim between once a week (338/996, 34.0%) and a few times per week (418/996, 42.0%). Any selection of ‘Do not swim in the winter’ was excluded from the analysis.

**Figure 8.**
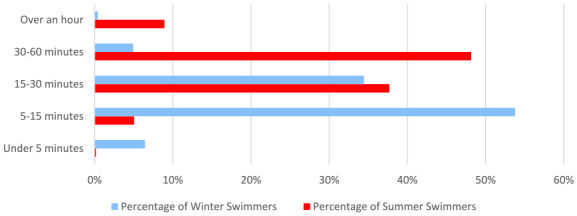
A comparison of how long women swim for on average, in the summer versus the winter.

Women were asked how long these swims would last (Figure 8). In the summer, the most common length of time for a swim was 30–60 min (534/1109, 48.2%). In the winter, the majority of swims were stated to last between 5–15 min (534/993, 53.8%).

**Figure 9.**
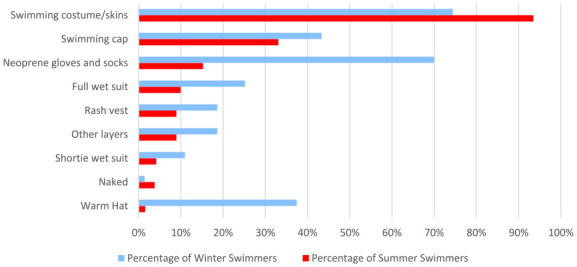
A comparison of what women wear to cold water swim in the summer versus the winter.

Women were asked what they wear while swimming, selecting all answers that applied (Figure 9). The most common item worn in the summer in this cohort of women is swimming costumes/skins (1037/1109, 93.5%). Those who selected ‘Do not swim in the summer’ were excluded from this analysis. In the winter, the majority of women stated they wear swimming costumes/skins (739/993, 74.4%), but a considerably higher percentage stated they also wore neoprene gloves and socks (695/993, 70.0%). Those who selected ‘Do not swim in the winter’ were excluded from this analysis. The percentage of women who wear a wetsuit increased in winter (250/993, 25.2%) compared with summer (110/1109, 9.9%).

### Free-text comments

At the end of the survey, 745 women gave free-text comments, with the majority stating that cold water swimming helped their mental and physical health and that being in nature and swimming with friends made them happy. A sample of these are reported.

*Cold water swimming has been so good for my mental health. . . . .The elation after a swim is remarkable and priceless.*

*Certainly puts you in a good mood and sets you up for the day*

*The main gain for me is it resets my mood if I’m feeling stressed or anxious.*

*I feel cold water swimming helps my emotional health and clears my head. I do feel physically better when I have gone for a swim.*

*Open water swimming is my happy place.*

*Wild swimming completely resets my physical and mental state to calm.*

*I like being in the fresh air and the effect of the invigorating sea is beneficial to mental health*

*Swimming outdoors gives me a sense of wellbeing because I am free to swim when I want, where I want and for how long. Immersing myself in a loch where there are no people, kids, lanes is just bliss.*

*I feel I have stolen a moment that nobody else will have. I feel alive and thankful.*

*Lifts your mood, you are with nature and the sea. It’s relaxing and so much fun. Going with a female friend is just the best. Makes you feel SO ALIVE!*

*If I am unable to swim for more than a week, my mental health deteriorates.*

*Cold water is phenomenal. It has saved my life. In the water, I can do anything.*

*Leave my worries on the sand and for the time I’m in the sea I feel me again*

*Exercising in nature, alone or with a group of other women is healing.*

## Discussion

Cold water swimming has become a highly popular sport but this is the first study specifically asking about women’s cold water swimming habits. In this survey, we found that women’s swimming habits varied, but most of the women were likely to swim in both summer and winter, wearing swimming costume/skins all year-round and most swim for 30–60 min in the summer which reduces to 5–15 min in winter. As well as a reduction in swim time in winter, many added neoprene gloves and boots to keep warm and some moved to wearing a wetsuit, which would reduce the rate of local muscle cooling and deep body cooling. This enabled them to continue swimming even during the coldest months. But it is important to note that swimming in the UK winter for prolonged time gives an increased risk of hypothermia,^27^ peripheral neuromuscular cooling and incapacitation.^25,26^

### Benefits of cold water swimming

In our study, the reasons for swimming varied but the majority do so to be outside, to support recovery from poor mental health or maintain good mental health and exercise. The positive effects of exercising outside, often with a community, and in cold water, were reported to have health benefits and align with previous studies.^1,21^ Women were most likely to swim with other people, which could be for safety reasons but may also indicate the importance of community. Social isolation is an important determinant of mental and physical health so this is likely to contribute to the well-being benefits derived from the activity.^32^

A set of data from the same survey that was reported here asked women if they felt cold water swimming affected their menstrual and menopause symptoms.^31^ Women reported that cold water swimming reduced their menstrual symptoms: notably, psychological symptoms such as anxiety (46.7%), mood swings (37.7%) and irritability (37.6%). Perimenopausal women reported a significant improvement in anxiety (46.9%), mood swings (34.5%), low mood (31.1%) and hot flushes (30.3%). The majority of women (56.4% for period and 63.3% for perimenopause symptoms) swim to relieve these symptoms and they felt that symptoms were helped by the physical and mental effects of the cold water which was more pronounced when it was colder. How often they swam, how long for and what they wore, was also important.

The majority of women in this survey swim in the wild: sea, lakes, rivers and reservoirs, with only 12% swimming in an outdoor pool. The free-text question corroborated this, with many of the women commenting on the freeing and life-affirming impact of being in a natural space. The importance of ‘green’ and ‘blue spaces’ for overall health may be a key benefit of cold water swimming.^33,34^ An Australian study suggested the additive effect of exercising in the blue space, contributing to a reported improvement in well-being.^35^ This provides a basis for the encouragement of cold water swimming, rather than CWIs like cold showers, which may not elicit the same positive effect due to the lack of ‘blue therapy’.^10^ But there are other types of exercise that can be done in the water, outside, such as surfing body-boarding, and sea hiking, which may well have the same positive effects.

### Barriers to cold water swimming

Despite the many benefits reported, swimming in cold water is not without risk. In the United Kingdom, the government bathing water quality website only runs from May–September, ignoring the winter months where heavy rainfall and consequent sewage overflow most commonly occur.^36^ In addition, cold water swimming is dangerous, particularly for novices. The risk of hypothermia and ‘immersion pulmonary oedema or swimming induced pulmonary oedema’ is not insignificant.^8,28^

There may be social barriers to the uptake of cold water swimming, notably those who cannot swim. The Sport England Report showed 95% of Black adults do not know how to swim.^37^ There are a number of barriers for people who have no experience of swimming, particularly people from ethnically diverse backgrounds, as many swimming pools can be expensive to access, can be intimidating mainly white spaces. Therefore, the group of people from ethnically diverse communities who may wish to swim outdoors is at present small. To increase diversity in outdoor swimming, we need first to develop spaces that welcome diversity and support ethnically diverse communities to participate in swimming.

In addition, economic barriers may play a role. Although considered a ‘free activity’, cold water swimming can come with costs: being close to somewhere to swim, access to open water (which is supported in Scotland but not in the rest of the United Kingdom), access for those with disabilities, parking, equipment and kit required, as well as the time taken to do the activity. These factors make cold water swimming less accessible than previously theorized.

### Study limitations

The limitations of this study must be recognized. A power calculation was not done. We aimed to get as many respondents to the survey as we could. By using an online survey to conduct our research, there is no way to verify the participants met our inclusion criteria, and therefore, the results may be misaligned with that of the true population. Furthermore, the use of an online survey introduces a sampling bias, where those without access to the Facebook groups or the social media sites used would not have been able to fill in our survey.^38^ The study is also limited by the demographics of the sample: mostly representative of White, highly educated women as highly educated and/or White women are more likely to fill in surveys^39^ which may have been limited by the social media sites the survey was advertised on.

## Conclusion

The women who cold water swim have reported the physical and mental health benefits from swimming. Exercising in nature, with a community, is a combination that should be encouraged. But cold water swimming has the added benefit of the effect of the cold water, which many women said helped their physical and mental health. It was not surprising to that women swim for longer in the summer than in winter, and they wear more clothes in winter.

Globally, we should be ensuring that cold water swimming is accessible and safe. In England and Wales, cold water swimming is restricted, with a lack of support for cold water swimmers, especially with access to swim spots, access for those with disabilities and the issue of pollution. Scotland encourages cold water swimming, as swimmers have a right to swim as part of their statutory right of responsible access to most land and inland water. It is time to make cold water swimming safer and more supported.

## Supplemental Material

sj-docx-1-whe-10.1177_17455057241265080 – Supplemental material for The swimming habits of women who cold water swim Supplemental material, sj-docx-1-whe-10.1177_17455057241265080 for The swimming habits of women who cold water swim by Megan Pound, Heather Massey, Sasha Roseneil, Ruth Williamson, Mark Harper, Mike Tipton, Jill Shawe, Malika Felton and Joyce Harper in Women’s Health

sj-docx-2-whe-10.1177_17455057241265080 – Supplemental material for The swimming habits of women who cold water swim Supplemental material, sj-docx-2-whe-10.1177_17455057241265080 for The swimming habits of women who cold water swim by Megan Pound, Heather Massey, Sasha Roseneil, Ruth Williamson, Mark Harper, Mike Tipton, Jill Shawe, Malika Felton and Joyce Harper in Women’s Health

## Declarations

**Ethics approval and consent to participate:** This project was approved by UCL Research Ethics Committee: 9831/007. To undertake the survey, participants had to click consent to continue onto the survey questions.

**Consent for publication:** Agreed.

**Availability of data and materials:** Some anonymous data may be available after consultation with the UCL ethics committee.
